# “*When we stop choosing them*.” Recovering freedom in intimate relationships among adolescents

**DOI:** 10.3389/fsoc.2025.1685768

**Published:** 2026-01-05

**Authors:** Marifa Salceda, Ana Vidu

**Affiliations:** 1 Department of Education, School of Education, University of Cantabria, Santander, Spain; 2 Department of Private Law, School of Law, University of Deusto, Bilbao, Spain

**Keywords:** preventive socialization of gender-based violence, coercive discourse, violence, intimate relationships, dialogic gatherings

## Abstract

Rising violence in adolescent sexual-affective relationships is a global concern. Studies attribute the issue to socialization towards violence and coercive discourse, which might contribute to pushing girls into violent and non-egalitarian relationships. Prevention hinges on science-backed dialogic interventions, focusing on attraction and election of egalitarian relationships. This research examines a dialogic gathering intervention with 15 heterosexual adolescent girls (aged 15–18), mainly using the book “Radical Love.” Communicative Methodology assessed the impact on the participants, with audio-recorded dialogues, life histories, and focus groups. Results reveal the dialogic gatherings’ effectiveness in countering coercive discourse and empowering participants to freely choose the safest and healthiest sexual-affective relationships.

## 1 Introduction

The World Health Organization considers Gender-Based Violence (GBV) to be a violation of human rights and a public health issue due to its prevalence and global reach (World Health Organization, 2013). According to the Committee on the Elimination of Discrimination Against Women (CEDAW), this is a form of discrimination that disproportionately affects women simply because they are women, prevents them from enjoying their rights and freedoms on an equal basis with men, and includes acts that involve physical, mental, or sexual harm or suffering, threats, harassment, coercion, and other forms of deprivation of liberty (CEDAW, 1992). In 2015, the Spanish Government Delegation against Gender-Based Violence published a report exploring adolescents’ perceptions of this issue, including a specific chapter on its prevalence among young people. The findings of this study, included in the 2015 Spanish report mentioned, revealed that 29% of adolescents were aware of a case of gender-based violence in their close environment, and in 21% of these cases, the victim was a minor (Luken, 2015). Another study analyzing changes in gender equality attitudes and prevention of GBV among Spanish adolescents indicated a slight decline in the justification of such violence compared with the diagnosis obtained in 2010 (Díaz-Aguado et al., 2014). Nonetheless, the results remain concerning, particularly due to the increase in adolescents reporting experiences of abuse, including control, insults, humiliation, fear, sexual coercion, physical aggression, and online defamation from their partners. There was also a notable rise in boys admitting to perpetrating this violence.

In the same study, 7.8% of adolescent girls agreed with the statement “the man who seems aggressive is more attractive” (Díaz-Aguado et al., 2014, 102). This, along with other insights into adolescent sexual-affective relationships, points to an urgent need for enhanced efforts in education, awareness, and prevention. Worryingly, physical appearance has become increasingly prioritized among adolescent girls, while values like kindness and sincerity have lost prominence (Díaz-Aguado et al., 2014).

Considering the results of the latest macro-survey on violence against women in Spain, the situation is not improving. Young women aged 18 to 24 report the highest rates of psychological violence (control by their male partners) in relationships and sexual violence outside them, with those aged 16 to 24 particularly affected. These trends may reflect both greater exposure to violence and increased recognition and willingness to speak out about it (Spanish Government, 2021, Violence against women macro-survey 2019). Being young (especially for girls aged 16 to 24) is now considered a risk factor, with higher levels of reported psychological abuse by boys in intimate relationships than in any other age group (Díaz-Aguado et al., 2014). This disturbing trend underscores the need for more scientific research to develop effective strategies to combat GBV.

Additionally, current studies identify a dominant coercive discourse in society that influences many adolescents, boys and girls, to enter or remain in violent relationships (Pérez-Martínez et al., 2023; Racionero-Plaza et al., 2021; Ruiz-Eugenio et al., 2020). Further research has explored how socialization processes throughout life shape which traits are considered attractive, often linking violence with desirability (Torras-Gómez et al., 2022; Ugalde et al., 2022).

Dialogic reading, one of the most effective Successful Educational Actions, has been proven to enhance learning, emotional well-being, and social cohesion across various contexts (European Commission, 2006-2011). Further research has also shown how dialogic gatherings help reveal and challenge the coercive discourse by equipping young women and minors with tools to recognize and combat it, ultimately supporting the pursuit of healthy, non-violent relationships (Allotey et al., 2023).

Originally implemented at the La Verneda-Sant Martí Adult School in Barcelona (Sánchez-Aroca, 1999), dialogic gatherings have since expanded across all educational levels - including mainstream and special education (Fernández-Villardón et al., 2021; Ruiz-Eugenio et al., 2023) - as well as into settings like residential care for minors (García-Yeste et al., 2018), prisons (Álvarez et al., 2018) and mental health programs (Fernández-Villardón et al., 2021). These gatherings not only foster intellectual growth but also improve personal well-being and interpersonal dynamics (Ruiz-Eugenio et al., 2023).

## 2 The social theory of heterosexual affective relationships

Socialization is the lifelong process through which people internalize norms, values, and behaviors, beginning in childhood and shaped by those around us. Dialogue and social interaction are the foundation of all knowledge and the essence of what makes us humans (Giddens, 1982; Puigvert et al., 2019). Individual thoughts and attitudes emerge socially, developing through language and interpersonal exchanges within a cultural context (Olave, 2023; Vygotsky, 2015). As Mead (1967) emphasized, the individual cannot exist independently from social experience.

This perspective offers a hopeful path toward eradicating gender-based violence (GBV). Since harmful relationship models are socially constructed, they can also be socially transformed. By reshaping how we are socialized into affective and sexual relationships, we can replace patterns rooted in domination and harm with ones grounded in mutual respect, equality, and emotional connection (Oliver and Valls, 2004).

Scientific research has not only highlighted the troubling prevalence of violence in adolescent relationships, particularly with girls as victims in sexual-affective relationships with boys (Adhia et al., 2019) but also contributed valuable insights into prevention and overcoming by delving into the social basis of GBV. These findings point to traditional notions of attraction and partner selection as key factors. Socialization processes that associate desire with violence play a central role in normalizing abuse and mistreatment in romantic relationships (Gross et al., 2006; Ríos and Christou, 2010).

### 2.1 The dominant coercive discourse linking attraction and violence

Research on early childhood violence prevention highlights a troubling pattern: many boys who display aggressive behavior are not marginalized but instead attain positions of social power (Cañas et al., 2022). These boys often exhibit strong interpersonal skills and are viewed as socially appealing (Hawley et al., 2007; Cheng et al., 2021; Rodríguez-Oramas et al., 2020). As Slaby (1995), they tend to attract adult attention more easily than children who are quietly engaged in cooperative or constructive play. Neglecting how violence is learned through socialization -from childhood onward- has serious consequences. It can lead us to tolerate harmful behaviors in romantic contexts that we would readily reject in friendships (Hooks, 2018). Framing love as a matter of biology, chemistry, or fate rather than social construction reinforces the idea that relationships are outside our control, as if governed by instinct or “destiny” (Beck and Beck-Gernsheim, 2001). This narrative helps justify and even glamorize toxic relationships, portraying them as thrilling and inevitable, while undermining our agency to choose and change how we relate to others (Lelaurain et al., 2021).

Messages that reinforce GBV are often transmitted to minors and adolescents through romantic myths, especially in heterosexual relationships. These include the notion that love is irrational or accidental -something sparked by “Cupid”- as well as the glorification of “bad boys” with dominant traits and girls valued mainly for their appearance (Elboj-Saso et al., 2022; Padrós et al., 2010). Research shows a strong association between attraction and violent behavior in heterosexual relationships where traditional dominant masculinity is idealized (Duque, 2008). In these researches, conversely, ethical or egalitarian male partners are frequently viewed as suitable but lacking passion, resulting in a resigned acceptance of loveless “good boy” relationships (Gómez, 2015). This form of socialization is absorbed not only through family, schools, and peer groups but also through powerful media influences (Villarejo-Carballido et al., 2022). Teen magazines focused on heterosexual girls often promote rivalry among girls, treat relationships as tools for status, and equate attraction towards men with the forbidden or difficult (Gómez, 2015). Similarly, best-selling books with poor literary value that are adapted into films featuring popular actors, violent video games, music with aggressive lyrics, and reality TV shows all play a role in reinforcing to heterosexual females violent and unequal models of attraction and masculinity (Gabbiadini et al., 2016).

### 2.2 Dialogic gatherings: communicative action for socialization in egalitarian models

Women who experience GBV often lack safe spaces where they can openly share their experiences and receive support (Chung, 2007; Gómez-González et al., 2022). Dialogic gatherings designed to prevent and overcome GBV offer such spaces, where participants engage in conversations grounded in scientific evidence on the everyday dynamics that act as risk factors. These exchanges enhance participants’ awareness of violence and its roots, encourage egalitarian communication (Pick et al., 2010) and support more autonomous and informed decisions regarding all types of intimate relationships.

Research has extensively explored the tension between the language of ethics and the language of desire (de Flecha-Fernánz Sanmamed and Puigvert, 2010). From a heterosexual perspective, this divide often surfaces in how adolescents communicate, where egalitarian men are ridiculed, and attraction is frequently directed toward violent masculinities (Bukowski et al., 2000; Zubiri-Esnaola et al., 2021). Dialogic gatherings address these dynamics through communicative acts that encourage participants to compare personal experiences with scientific knowledge on violence, fostering what Kitzinger (1994) termed “complementary interactions” and prompting re-evaluation through “argumentative interactions.” These exchanges help reshape previously held beliefs that may perpetuate harmful socialization.

This process not only deepens understanding but also ensures that interpretations are grounded in scientifically validated arguments, drawing attention to the broader social and contextual factors at play (Aubert et al., 2011). As a result, these dialogues support a model of attraction where kindness, desirability, and passion coexist (Gómez, 2015). Reflecting intersubjectively on one’s own socialization contributes significantly to the recovery of survivors of GBV (Melgar et al., 2021). To dismantle the communicative acts of power that are reinforced through peer influence, social status, and societal pressure to pursue certain relationships (Ríos and Christou, 2010), the alternative model of sexual-affective relationships emphasizes the role of dialogic interactions in any relationship. These are characterized by mutual respect, honesty, and solidarity, and they aim to prevent or overcome violence through collective reflection. Such dialogue has proven effective in challenging the appeal of violent behaviors and instead promoting traits like empathy, safety, and kindness as attractive. This shift contributes to reposition non-violent individuals as desirable, while reducing the attraction of those who may exhibit aggression (Duque et al., 2015).

## 3 Methods

This study was developed through the Communicative Methodology (CM) (Gómez-González et al., 2019). This approach was chosen because the research process introduces transformations for preventing gender-based violence by allowing an egalitarian dialogue in which the researcher provides contributions from the existing scientific literature. This continuous process triggers reflections resulting from the interaction between all participants, which encourages the critical review of previous ideas related to violence in sexual-affective relationships and how to overcome it (Pulido et al., 2014).

All research focuses on social impact, and therefore, the postulates of the CM developed by Gómez-González et al. (2019) have been taken into account in each intervention as described below. Firstly, the *universality of language and action*, maintains high expectations for the development of communicative and critical thinking skills of all participants. Secondly, considering the *adolescents in care as transformative social agents*, capable of critically understanding reality in order to transform it. Thirdly, taking *communicative rationality* as the axis guarantees that in each meeting, the dialogue with the participants will take place under equal conditions for the collective construction of knowledge. Fourth, bearing in mind that the participants’ *common sense* is shaped by the interactions and the context in which they take place. Therefore, both the intervention and the data collection took place in the residential care setting where the participants live, and this facilitated a safe and trusting environment. The fifth postulate of the CM is the *abolition of the interpretative hierarchy*. Throughout the research process, the participants’ ability to interpret their own reality has been the starting point, while improving collectively through the arguments shared at each meeting. This implies that the research team has not monopolised knowledge but has contributed to bringing scientific knowledge closer together without holding a position of power. Sixth, an *equal epistemological level* between researchers and participants has been intended. This implied that the knowledge and experiences shared by the participants have not been instrumentalised, but that the dialogue has been enhanced by contributing the proper scientific knowledge and combining it with the knowledge of the participants. Finally, the *dialogic knowledge* has been considered to reflect, promote interactions and generate new knowledge based on inter-subjectivity, egalitarian dialogue, solidarity and consensus.

### 3.1 Participants

The sampling procedure of participants in this study comprised 15 heterosexual adolescent girls coexisting in a group home in Spain due to causes associated with different types of gender-based violence. We did not directly choose the girls; we just wanted to analyze a sheltered apartment functioning in a rather small city in Spain. We have been contacted by the social worker (appointed by the government to oversee the apartment and the girls living there) to participate in this plan they had, to read a book once a month in a gathering format. We helped organize the sessions using the described methodology. We had no recruitment criteria for the adolescent girls. We did not include or exclude anyone. We did not ask for their sexual orientation. Their number varied slightly throughout the research, as not all girls attended all interventions. It was not mandatory for them to attend, even though most of them liked the sessions and never missed any. In case of dropouts, we carried on with the session as usual. The group, ranging in age from 15–18 years, was diverse in terms of ethnicity, culture, education, and socioeconomic background. The common denominator was that they were under an institutional protection system and were survivors of violence.

### 3.2 Ethics statement

The fieldwork has been developed with a sample of vulnerable populations because they are adolescents in care, who incorporate family separation measures, and are survivors of violence. Therefore, the study followed all ethical standards for research, the 2001 Code of Ethics from the International Sociological Association (2025), the 2019 Ethical Guides from the European Sociological Association (2025), and the 2010 European Textbook in European Commission (2025). The security, anonymity, and privacy of all individuals involved in data collection and analysis were ensured. The purpose and objectives of the research were explained, as well as the voluntariness to participate or to stop doing so at any time. Participants and legal guardians completed written informed consent forms.

### 3.3 Procedure

The procedure for conducting a dialogic gathering is as follows. Beforehand, participants read the agreed part of the book for each session and choose a paragraph or an idea to share with the group on the day of the meeting. In each session, the participants shared a section from the book and discussed ideas that connected to their lives, generating an equal and respectful dialogue (Ruiz-Eugenio et al., 2023; Salceda et al., 2022). For this reason, an essential condition is that the chosen readings are highly scientific or universally recognized for their literary quality (Keidel et al., 2013). This collective construction of knowledge from literary works of the highest quality generates opportunities to reflect on one’s life trajectory and to draw better horizons (García-Yeste et al., 2017). The social worker had no role in the intervention. They only had an observer role. We also noticed that the girls were not limited in their participation because of the social worker’s presence; rather the opposite, they feel confident and in a trusting environment.

### 3.4 Intervention

The research involved 14 dialogic gatherings focused on the evidence of the research line of preventive socialization of gender-based violence. Half of the interventions, the first seven, were carried out through the book *Radical Love* (Gómez, 2015), for which there is strong evidence of its contribution to the preventive socialization of GBV in adolescence (Ugalde et al., 2022). This is an academic text on socialization and love that links the dominant coercive discourse with victimization due to gender-based violence. Its selection was based on previous research on dialogues surrounding its content, which have proven effective in supporting the free reconstruction of memories of violent sexual-affective relationships that can lead to safer future choices of partners, whether stable or sporadic (Racionero-Plaza et al., 2018).

For the other half of the interventions, the group chose three books considered universal literature because of their literary quality, which allowed them to delve deeper into the theme of current sexual-affective relationships based on the characters’ life stories and to transform their visions towards the ideal of love; sharing ideas, doubts, thoughts, and personal experiences through reading and dialogue. These books were The House of Bernarda Alba (Lorca), Romeo and Juliet (Shakespeare), and The Metamorphosis (Kafka). Each of these stories allowed participants to connect personal experiences with their socialization on love and sexual-affective relationships, or how these can affect their mental health (Salceda et al., 2020).

Each intervention was conducted monthly; each dialogic gathering lasted 2 h and took place in the group home with the participation of a social worker and two researchers (the authors). For each meeting, the social worker and the researchers read the agreed part of the book in addition to the participants. This process was carried out carefully and by the Communicative Methodology, which allows researchers and participants to attend and intervene on equal terms in generating new knowledge, eliminating interpretative hierarchies, and considering all people on the same epistemological plane (Gómez-González et al., 2019). Even trying to achieve an equalitarian dialogue, −which is encouraged by CM-, during the interventions, we were aware of our role as researchers and the potential influence we may have on the participants, even if we are of the same race and gender as them. To compensate for this reality, during the gatherings we refrained from commenting on their interventions, from judging or analyzing their thoughts.

### 3.5 Measures and instruments

The dialogic gatherings were audio-recorded, transcribed verbatim, and supplemented with observations and field notebooks. Two life histories and two focus groups with a communicative approach were also conducted, one before and one after the intervention. The life histories were carried out at the end of the intervention, holding a dialogue with the participants to reflect on the impact of these dialogic gatherings on their thoughts, relationships, and lives. In the dialogic gatherings, the researchers introduced the most relevant theories and scientific research in the preventive socialization of gender-based violence into the topics of conversation in line with the readings. Through egalitarian dialogue, the focus groups allowed us a collective interpretation of the reality of coercive discourse in sexual-affective relationships by confronting individual and collective understandings.

### 3.6 Data analysis

Qualitative data were analyzed to identify adolescent girls’ perceptions of the impact of dialogic gatherings on their models of sexual-affective attraction and election and the prevention of coercive discourse linking attraction and violence. The results have been classified into three categories defined by consensus among the researchers and taking into account the scientific literature on the dominant coercive discourse and the preventive socialization of gender-based violence: (1) Identify the dominant coercive discourse that links attraction to violence in their tastes and sexual-affective relationships, (2) Recognize this dominant coercive discourse in the peer group in their environment, (3) Dialogue to overcome these barriers and recover a freer election of their sexual-affective relationships.

## 4 Results: overcoming the dominant coercive discourse

### 4.1 Attraction to violence: identify the dominant coercive discourse

Some adolescent girls become aware that they play a role in perpetuating traditional gender norms in relationships, primarily in two ways.

First, they reinforce conventional ideas of attractiveness associated with masculinity and femininity. For example, they admire stereotypical “ideal” couples, such as the most popular boy and the most stylish girl:

*"Look, we were in high school, and I envied the hot football player and the fashionable girl with the best body, who were together."* (Gina, 18 years old)

Second, they recognize a pattern of being attracted to rather aggressive behavior and how this leads to social support for boys who act violently:

*“The worse they treat you, the more hooked you get.”* (Isa, 17 years old).

This attraction persists even when they understand that such behavior reflects a toxic form of masculinity, lacking in egalitarian values that have already been violated previously:

*"I admit it, the guy in “Three Steps Above Heaven”* [film] *was a badass, but that’s why I liked him... because he’s badass. He is a beast, a brute, a macho guy—he’s like, the God of girls."* (Carmen, 17 years old)

However, as these conversations progress, some begin to question these attitudes, which makes this idea unattractive. What follows below is a dialogue between two teenagers and the social worker:

- *“That’s so tacky.”* (Fanny, 16 years old)
- *“Do you feel good knowing he hits girls?”* (Social Worker_1)
- *“I do not mean hitting girls. I just like him as a friend.”* (Carmen, 17 years old)
- *“Then find a friend who’s exciting and treats women well!”* (Fanny, 16 years old).

Failing to openly reject non-egalitarian relationships ends up making equal, respectful relationships seem less appealing. For example, one girl admits being drawn to bad treatment and not rejecting unequal relationships:

*"I get hooked on that, the more difficult. The worse he treats me, the more I’m into him."* (Gina, 18 years old)

At the same time, she dismisses respectful boys as weak or unattractive:

*"In high school, we call the guy who says, 'You’re beautiful' and treats me nicely a sucker."* (Gina, 18 years old)

Another girl echoes this same idea:

*"I have good-looking guy friends, and I used to wonder why they didn’t have girlfriends. Now I get it - girls go for the bad boys!"* (Carmen, 17 years old)

When decisions about romantic relationships are made without open dialogue or shared ethical values, they tend to follow outdated stereotypes of attraction. This disregards scientific understanding and moral considerations in the choice:

*"For boys, if girls are not pretty or hot, it doesn’t matter. And we’re supposed to like pimps and bad boys. That's enough. What matters is how they treat you. We should choose guys who treat us well and know how to respect us."* (Diana, 17 years old)

As these discussions continue, at each dialogic gathering, more girls begin to express strong criticism of traditional models of masculinity through their contributions to the discussion (Book 1, meeting 3). They start to better identify signs of inequality and emotional harm and understand the value of honest conversations to change these dynamics:

*"I’d never thought about it like that, but it’s true. They don't enjoy themselves and they’re not enjoying the relationship; they’re just out to hunt. It makes you feel like an object. With guys like that, you get into a toxic cycle…"* (Ada, 15 years old).

*"Yeah, they want you as a prize. They chase you, use you, then toss you aside."* (Diana, 17 years old).

### 4.2 Recognizing coercive discourse in the peer group

Adolescents explain that coercive discourse (understood as social pressure that influences romantic and sexual choices) often comes from their peer group. This group exerts a strong influence over whom they find attractive and choose to date. When asked why they are drawn to certain types of masculinity, one girl answered:

*“So that your friends will think he looks good.”* (Henar, 15 years old).

Carmen (17 years old) supported this, saying:

“*Exactly, like she said. My friends ask if he’s good-looking, not if he has values*.”

Taking control over one’s romantic choices becomes especially important when the coercive pressure that encourages attraction to violent or dominant behavior has not yet been addressed. One girl reflects on this after reading a passage from *Radical Love:*

*“I chose this part... about how irrational we are when we choose a boyfriend, because it’s true, it happens to me (...). You get hooked on that person. It’s like smoking; we know it may kill us, but we still do it. When really, we should choose someone who brings something good into our lives.”* (Elsa, 17 years old)

When romantic choices remain trapped within this coercive discourse, the outcomes tend to be the same: emotional suffering, dissatisfaction, and a sense of hopelessness. Gina (18 years old) shares a personal experience:

“He told me, ‘I don’t have girlfriends, I have girls I hook up with,’ like… I already knew what that meant … and Fanny or Ali had warned me too. But I didn’t want to believe it, I wanted to think he was the perfect guy. But no! It’s always the same.”

This story shows that Gina was aware from the beginning that the relationship might not be fulfilling. Her doubt was based on (1) her own past experiences with similar types of boys; (2) warnings from her friends in the discussion group; (3) the boy’s own words, making his intentions clear. Despite all this, she still chose to believe in a different outcome, ultimately giving in to the coercive discourse that equates attractiveness with harmful masculine behaviors.

### 4.3 Recover freer election of their sexual-affective relationships

This category of analysis highlights two key aspects: a growing critical awareness among adolescents of the coercive discourse that shapes their romantic choices, and the power of dialogue to challenge and transform those patterns. On one hand, adolescents show they are starting to reflect critically on the social pressure that links attraction to harmful models of masculinity, and some are actively rejecting it:

*“At least you realize it. That’s the key step, the most important one.”* (Fanny, 16 years old).

This awareness is often sparked through engagement with literature and thoughtful discussion:

- *“Yeah … I do not know, it’s incredible. What happens in the story is real.”* (Julia, 18 years old, referring to *The House of Bernarda Alba* by Federico García Lorca). *I do not know; I think these are the kind of books that are worth it* (…).
- *“This is better than After.”* (Ada, 15 years old, referring to a popular book and film series, which is trendy among teenagers, that romanticizes violent relationships)
- *“It helps you put yourself in their place, and… it changes how you think.”* (Kiara, 18 years old).

On the other hand, dialogic gatherings (conversations based on respect, evidence, and shared reflection) create a space where adolescents can begin to free themselves from these coercive social norms. Through these discussions, they come to understand that love and friendship must not involve pain or harm.

*“If it hurts, it’s not love or friendship or anything like that.”* (Carmen, 17 years old).

Fanny (16 years old) reflects on an excerpt from *Radical Love* book and points to the danger of media that glamorize emotional detachment and disrespect in relationships:

“They promote deceit and a lack of respect… We can’t act like that’s normal—because it isn’t.”

Group dialogue and reflection also help adolescents reevaluate past intimate experiences that were not based on egalitarian values. The support they receive in the group makes it possible to reject deceit and infidelity openly. For example, Carmen once shared that she had been cheated on in a relationship but had “freely” chosen to forgive the betrayal. However, after participating in six dialogic gatherings, her understanding of the situation changed significantly, and she even explains it differently. She was no longer trapped by the narrative that normalized inequality and deception:

*“I haven’t been through that. Because when someone hurts me, they push me away (…). If you hurt me once, I know it won’t be the last time.”* (Carmen, 17 years old)

The ability to freely choose whom to love, without pressure or distortion, is seen as central to preventing violence and disappointment in relationships:

*“That’s what hurt so much - thinking you could have an ideal relationship, while choosing someone who didn’t offer equality. Of course, it was never going to work, not achieving that ideal, not with that person.”* (Sonia, 16 years old)

In fact, during the intervention, two girls admitted they were in non-egalitarian intimate relationships due to being influenced by coercive discourses about what kind of boys they should like. Thanks to the reflections sparked in the group sessions and the discussions about being free to choose, both decided to end those relationships. Their realization was clear:

- *“They’re the pimps, the bullies, the bad guys … This has caught my attention because it actually happens in my class.”* (Henar, 15 years old)
- *“And when do you think those boys will change?”* (Researcher)
- *When we stop choosing them* (Ada, 15 years old).

## 5 Discussion and conclusions

The language we use is never neutral; our words significantly shape the nature of our relationships (Ugalde et al., 2022). Habermas (2010) theory of communicative action, when applied to the prevention of GBV, underscores the importance of dialogue for fostering understanding in intimate relationships. Communication, in this context, is not about asserting power but about expressing thoughts, feelings, and desires with a claim to validity (Gómez, 2015). Research also reveals that particular expressions are often associated with unequal or non-egalitarian relationships, categorized within the realm of “what is exciting” (the language of desire). On the other side, individuals who embody values like kindness and equality tend to be described using terms from the “what is good for me” framework (language of ethics) (de López Aguileta et al., 2020). These dynamics are part of a coercive discourse that links attraction with violence in sexual-affective contexts (Aubert et al., 2011).

Aligned with prior studies, this research shows that dialogic gatherings create safe, supportive environments where participants (heterosexual adolescent girls) can openly reflect on GBV as it manifests in their everyday lives (Racionero-Plaza et al., 2018, 2021; Salceda et al., 2022; Ugalde et al., 2022). By consistently comparing their socialization to scientific insights, participants gain tools for processing memories and reinterpreting past relationships. This process serves not only as a mechanism for healing but also as a protective factor for the future (Melgar et al., 2021). Each dialogic gathering is shaped collectively, fostering a space of mutual trust and equality. Participants get to know one another more deeply, build trust, and feel comfortable discussing sensitive topics or seeking help in potentially risky situations. These exchanges often continue beyond the sessions, creating informal support networks that counter the dominant coercive discourse and help reclaim freedom in choosing sexual-affective partners.

The findings further support existing research showing that Communicative Methodology helps foster more egalitarian relationships (Gómez-González et al., 2019; Puigvert, 2016), considering heterosexual dynamics. Some girls, through their involvement, chose to leave harmful relationships with boys and transform their interactions, gaining tools to make more thoughtful and autonomous decisions about the people in their social lives. At the beginning of the research, some adolescents who had previously experienced GBV expressed a discourse shaped by victimhood and exclusion. The dialogic gatherings provided a platform for reshaping these narratives, supported by scientific knowledge and egalitarian interactions. This process helped participants regain agency and see themselves as protagonists of their own transformation (Melgar et al., 2021). Integrating scientific texts and literary narratives with personal experiences enabled collective reflection on attraction and choice, often leading participants to reassess normalized violence and consider either transforming or ending intimate relationships that conflicted with egalitarian values (Aubert et al., 2011; de López Aguileta et al., 2020; Racionero-Plaza et al., 2018, 2021). We have not found in our research that aspects such as diversity, social class, or cultural differences have any impact on the results achieved.

Traditional models of attraction and election in intimate heterosexual relationships deny the transformative power of dialogue and social interactions, reinforcing a socialization in which individuals replicate harmful cultural norms rather than actively shaping their lives. Katz (2008) highlights how dominant representations of aggressive masculinity tend to be symbolically reinforced in society. This study adds further evidence that intersubjective dialogue can disrupt those patterns and contribute to meaningful change. Nowadays, dialogic gatherings serve as both a preventive and protective measure, offering an effective way to transform the socialization of attraction and intimate relationships in the analyzed cases. As scientific understanding evolves, it is vital to ensure that prevention programs targeting young people are grounded in rigorous, evidence-based research. Continued inquiry with tangible social impact is essential to both prevent and overcome violence in heterosexual affective relationships from an early age.

As Gómez (2015) puts it, abuse could persist as long as abusive men remain more socially successful in heterosexual romantic contexts than those who embody respect and equality. One adolescent participant captured this idea powerfully: overcoming coercive discourse and reclaiming freedom in romantic relationships becomes possible “*when we stop choosing them*” (Ada, 15 years old).

## 6 Strengths and weaknesses

This study, like any research, has certain limitations. The first limitation concerns the sample size, which may restrict the ability to draw broad conclusions or identify strong correlations from the data. To address this, an extensive review of existing scientific literature on the preventive socialization of gender-based violence and dialogic reading was conducted. This literature review helped establish a solid foundation for understanding the research problem and enabled comparison of our findings with those of previous studies.

Secondly, this article focuses on a specific segment of a broader investigation involving dialogic gatherings, particularly those sessions where participants identified and discussed the dominant coercive discourse. It is important to clarify that dialogic gatherings are not used as therapeutic tools in this study; rather, they serve as a space for generating meaningful conversations. Evidence shows that discussions based on the world’s most significant literary works can have a profound educational and social impact. These include fostering personal growth, enhancing self-esteem, promoting non-violent environments, and building supportive relationships. In the context of gender-based violence, such gatherings also help participants develop the skills needed to recognize and overcome harmful relational dynamics.

In terms of limitations, the time available for data collection and analysis posed another limitation, making it difficult to assess long-term or sustained changes. Despite this, previous research has shown that the dialogues and interactions taking place in literary gatherings can lead to meaningful behavioral shifts. In this regard, we consider it relevant to consider as a future line of research the life stories of the participants, which could reflect the transformation processes in greater detail. In the study reported here, these sessions provided participants with a unique space to openly discuss - often for the first time - how the dominant coercive discourse might be affecting their lives. The reflective, intersubjective processes that emerged during each gathering equipped participants with critical tools to make more autonomous and informed decisions in their affective and sexual relationships.

Finally, we would like to mention the homogeneity of our sample as a limitation as well, which limits the extent to which the findings can be generalized to broader populations. Because the adolescent girls participating in the study shared similar characteristics in terms of sexual orientation, the observed effects may not fully capture the variability present in more diverse groups. It is possible that a more heterogeneous sample would show different patterns of responses or magnitudes of associations. Future research should incorporate greater diversity to assess the stability and applicability of these findings across a varied population.

## Data Availability

The original contributions presented in the study are included in the article/supplementary material, further inquiries can be directed to the corresponding author.
